# Thermal Boost Combined with Interstitial Brachytherapy in Early Breast Cancer Conserving Therapy—Initial Group Long-Term Clinical Results and Late Toxicity

**DOI:** 10.3390/jpm12091382

**Published:** 2022-08-26

**Authors:** Adam Chicheł, Wojciech Burchardt, Artur J. Chyrek, Grzegorz Bielęda

**Affiliations:** 1 Department of Brachytherapy, Greater Poland Cancer Center, 61-866 Poznan, Poland; 2 Department of Electroradiology, Poznan University of Medical Sciences, 61-866 Poznan, Poland; 3 Department of Medical Physics, Greater Poland Cancer Center, 61-866 Poznan, Poland

**Keywords:** thermal boost, hyperthermia, breast cancer, interstitial brachytherapy, conserving therapy

## Abstract

(1) In breast-conserving therapy (BCT), adjuvant radiation, including tumor bed boost, is mandatory. Safely delivered thermal boost (TB) based on radio-sensitizing interstitial microwave hyperthermia (MWHT) preceding standard high-dose-rate (HDR) brachytherapy (BT) boost has the potential for local control (LC) improvement. The study is to report the long-term results regarding LC, disease-free survival (DFS), overall survival (OS), toxicity, and cosmetic outcome (CO) of HDR-BT boost ± MWHT for early breast cancer (BC) patients treated with BCT. (2) In the years 2006 and 2007, 57 diverse stages and risk (IA-IIIA) BC patients were treated with BCT ± adjuvant chemotherapy followed by 42.5–50.0 Gy whole breast irradiation (WBI) and 10 Gy HDR-BT boost. Overall, 25 patients (group A; 43.9%) had a BT boost, and 32 (group B; 56.1%) had an additional pre-BT single session of interstitial MWHT on a tumor bed. Long-term LC, DFS, OS, CO, and late toxicity were evaluated. (3) Median follow-up was 94.8 months (range 1.1–185.5). LC was 55/57, or 96.5% (1 LR in each group). DFS was 48/57, or 84.2% (4 failures in group A, 5 in B). OS was 46/57, or 80.7% (6 deaths in group A, 5 in B). CO was excellent in 60%, good in 36%, and satisfactory in 4% (A), and in 53.1%, 34.4%, and 9.4% (B), respectively. One poor outcome was noted (B). Late toxicity as tumor bed hardening occurred in 19/57, or 33.3% of patients (9 in A, 10 in B). In one patient, grade 2 telangiectasia occurred (group A). All differences were statistically insignificant. (4) HDR-BT boost ± TB was feasible, well-tolerated, and highly locally effective. LC, DFS, and OS were equally distributed between the groups. Pre-BT MWHT did not increase rare late toxicity.

## 1. Introduction

The breast cancer treatment paradigm has changed continuously through the years, especially due to increasingly tailored approach to the patients. Surgery techniques alterations are clearly visible, neo- or adjuvant systemic treatment approaches rapidly evolve, and adjuvant radiation therapy methods and equipment improve as well. However, for most of early breast cancer (BC) patients, breast-conserving therapy (BCT) stays a standard treatment as an alternative to mastectomy. It demands conservative tumor resection, chemotherapy, modern immunotherapy and/or hormonal therapy depending on strict clinical indications. All followed by mandatory radiotherapy (external beam radiotherapy ± brachytherapy) [1,2,3,4,5].

At the time of the study, current trials proved that a higher dose administered to the tumor bed reduces 5-year local recurrence rate (LRR) from 7.3–13.3% to 3.6–6.3% [6,7,8]. When high-risk patients’ observation is prolonged to 20 years, it appears that boosting reduces ipsilateral breast tumor recurrence (IBTR) from 31% to 15% (*p* < 0.001) [9]. Concerning the high-dose-rate (HDR) brachytherapy (BT) boost technique, as used here, Polgar et al. summarized different HDR-BT protocols used in over 1770 patients, in whom boosting resulted in a mean 5-year LRR of 5.5% [10]. Local recurrences appear in the close surrounding of the resected gross tumor, and the cells there still have a recurrence-forming potential. The valid question is if there is such a treatment that would secure up to 100% local control (LC)? The place and importance of tumor bed boosting are still being researched worldwide [11]. 

One investigated option is augmenting the local irradiation efficacy with safely delivered thermal boost (TB) based on interstitial microwave hyperthermia (MWHT) preceding standard HDR-BT boost. Hyperthermia (HT) is well known and probably the most effective radiation sensitizer with large supportive data [12,13,14,15]. The most often underlined mechanism of cancer cells’ radio-sensitization is that controlled heat increase interferes with the cell’s proteins, especially those responsible for radiation-induced DNA structure damage repair [16,17]. The phenomenon is suggested to lead to tumor α/β ratio reduction [18].

Contemporary HDR-BT boost is based on temporary interstitial multicatheter implants, and CT imaging is standardly used for treatment planning [19]. The same implant and a set of CT scans can also be easily used for interstitial MWHT planning and its delivery shortly before the irradiation. However, the decision on implementing HT for a particular BC patient has to be personalized and based on a set of clinical features: breast size, tumor bed size and location, post-surgical breast presentation, sequelae (remnant seroma or hematoma), and interstitial implant volume [20].

This work aims to retrospectively report long-term results regarding the efficacy (LC; disease-free survival, DFS; overall survival, OS) and late toxicity of HDR brachytherapy with or without interstitial MWHT for selected early-stage BC patients treated with BCT. The paper is an update of previously presented short-term results [21] and an exhaustive extension of an interim conference communication [22]. HDR boost with or without the TB appeared to be equally and highly locally effective in the inhomogeneous BC patient groups. MWHT did not add to late toxicity. 

## 2. Materials and Methods

Between February 2006 and December 2007, 57 diverse stage and risk (IA-IIIA) BC patients were treated with BCT, and then standard adjuvant chemotherapy according to actual recommendations was administered. Surgery or both were followed by 42.5–50.0 Gy whole breast irradiation (WBI) and 10 Gy HDR-BT boost. Then, hormonal therapy was started if indicated. At the time of brachytherapy, after the tumor bed volume assessment and its relation to the rest of the treated breast tissue and covering skin, including post-implant coordinates and distances, patients were selected for additional pre-BT single MWHT treatment. Thus, the selection was not randomized and personally tailored. All qualified patients expressed individual informed consent for HDR-BT hyperthermic augmentation. Of note, hyperthermia provided combined with irradiation (interstitial brachytherapy included) is an accepted oncological treatment and reimbursed in the authors’ land. The last data update and further analyses were performed in January 2022.

The same interstitial implant was used for both treatments (BT and HT). Tiny microwave antennas with reliable thermometers (BSD-500 system operating at 915 MHz; BSD Medical Corporation, Salt Lake City, UT, USA) suit the flexible interstitial BT applicators well. Iridium-192 (^192^Ir) radioactive source (microSelectron-HDR, Nucletron BV/Elekta, Veenendaal, The Netherlands) was used for irradiation. Each HT session was planned to precede BT by 10–30 min, the reference temperature was set at 43 °C, and the intended therapeutic time (TT) of heating was one hour. Proper thermometry rules and obligatory quality assurance were followed after RTOG guidelines [23,24].

The decision to combine treatments was made based on a post-implant CT scans assessment. Thus, 25 patients (HDR-BT alone group, A; 43.9%) had a standard BT boost, and 32 patients (TB group, B; 56.1%) were additionally pre-heated. Refer to the patient and tumor characteristics listed in Table 1.

The interstitial BT technique was previously described in detail elsewhere [19]. Additionally, the report on the groups’ early toxicity and data on hyperthermia session measurements were already published in other series [21]. 

In the paper, both groups’ long-term clinical results (LC, DFS, and OS), CO and late toxicity (skin appearance, tumor bed hardening, and mammography findings) were retrospectively evaluated.

Data was collected in MS Excel, which was also used to obtain part of descriptive statistics. Further tests and figures were made using Statistica 13 (Statsoft, Tulsa, OK, USA). The *t*-test was used for continuous variables with normal spread. The Mann–Whitney test was used to compare categorical and continuous variables without normal spread. The variables in the nominative scale were assessed with a Chi-square test. OS, LC, and DFS were analyzed with the Kaplan–Meier method and compared with the log-rank test. The influence of selected variables on toxicity was analyzed with logistic regression analysis. The *p*-values below 0.05 were considered statistically significant. 

## 3. Results 

### 3.1. Clinical Results

**Figure 1:**
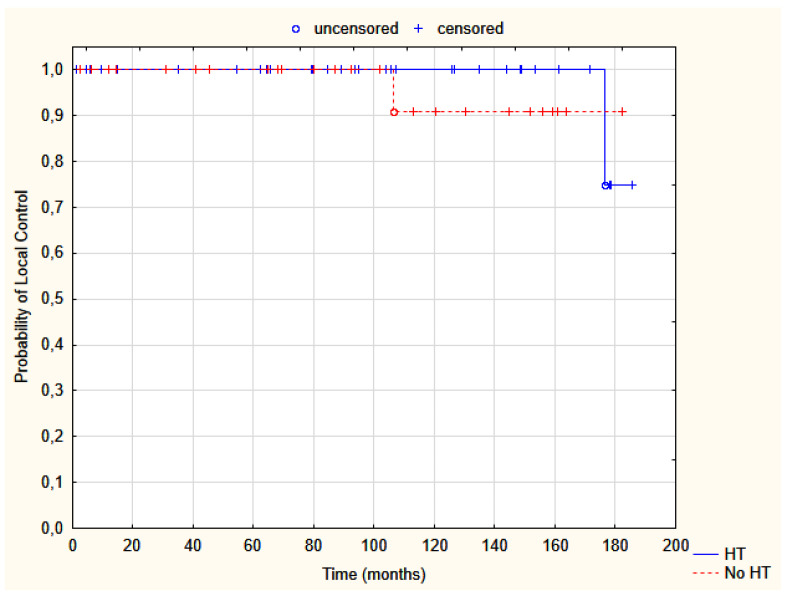
Probability of local control in group A, HDR-BT (red dotted line), and B, HDR-BT + thermal boost (blue solid line); Test log-rank, *p* = 0.59. HT—hyperthermia.

The median follow-up reached 94.8 months (range 1.1–185.5). During a median of nearly 8 years, only two patients developed local recurrences (LR) (crude LC was 55/57; 96.5%)—one in group A after 9 years and one in B after 15 years; both are alive (Table 2, Figure 1). The estimated 5-year and 10-year LC resulted in 100% and 96%, respectively.

**Figure 2:**
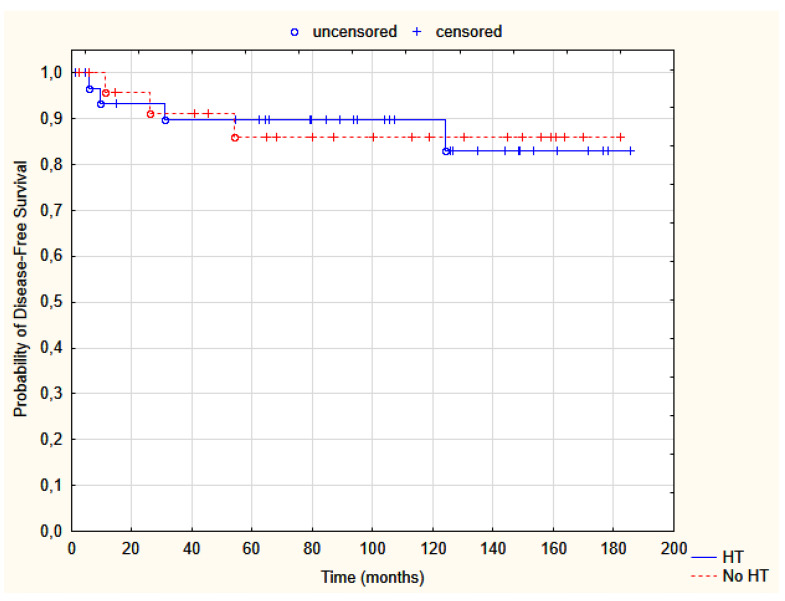
Probability of disease-free survival in group A, HDR-BT (red dotted line), and B, HDR-BT + thermal boost (solid blue line); Test log-rank, *p* = 0.98. HT—hyperthermia.

The 5-year and 10-year DFS was 88.3% and 88.3%, respectively. Overall, 7 out of 57 patients (12.3%) developed distant metastases (3 cases in group A, 4 in B) (Table 2). Of them, only one patient is still alive. DM and LR did not co-occur. Combined, DFS probability is displayed in Figure 2. 

**Figure 3:**
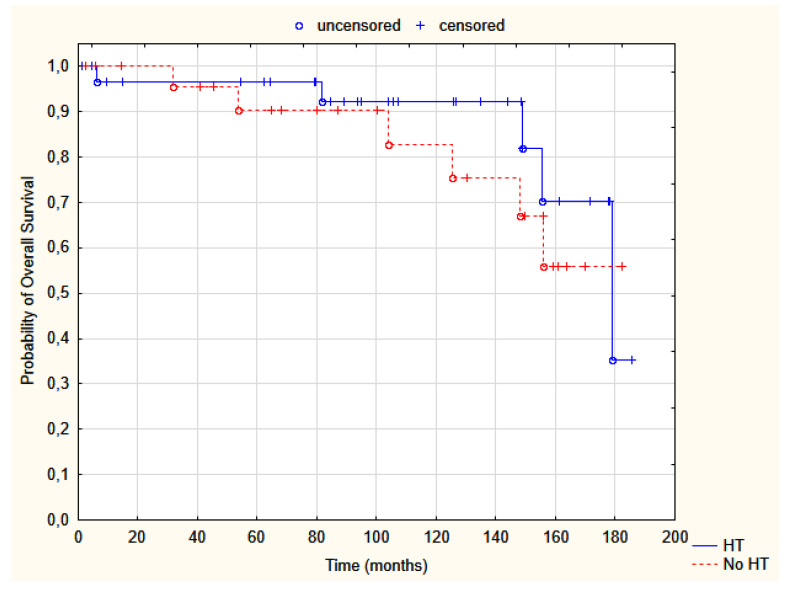
Probability of overall survival in group A, HDR-BT (red dotted line), and B, HDR-BT + thermal boost (solid blue line); Test log-rank, *p* = 0.46. HT—hyperthermia.

During follow-up, 11 patients died. The 5-year and 10-year OS was 94% and 88%, respectively. Six deaths were noted in group A and five in B (Table 2, Figure 3). All occurred after a mean time of 9 years, without LR. Six deaths were associated with BC distant metastases, and five had other causes (comorbidities—3; second malignancy—2). 

### 3.2. Cosmetic Outcome

CO assessed by consulting doctors according to Harvard criteria [25] was excellent in 60%, good in 36% (combined: 96%), and satisfactory in 4% in group A, and it was 53.1%, 34.4% (combined: 87.5%), and 9.4% in group B, respectively (Table 3). 

There was one identified outcome assessed as poor (group B). This particular case was investigated deeply. It was revealed that the treated breast was disfigured by surgery at the start. That caused increased WBI toxicity (RTOG grade 2) and challenging interstitial BT with TB, although feasible. In time, the patient developed massive breast fat necrosis with subsequent calcification. After about 10 years, it led to the formation of clearly palpable, more breast distorting, hardened, and painful mass. After 14 years post-BT, it was decided to perform mastectomy for symptom release (no tumor cell found in tumor bed). The patient has been BC free for 16 years now.

### 3.3. Late Toxicity

**Figure 4:**
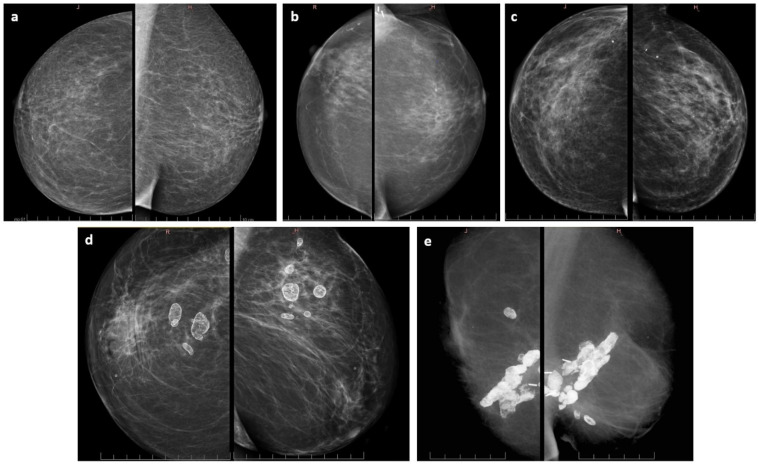
Control mammography findings (own material)—each left picture is a craniocaudal (CC) view. Each right image is the mediolateral oblique (MLO) view: (**a**) no secondary changes, (**b**) slight scarring of tumor bed, (**c**) scarring changes accompanied with dispersed and tiny benign calcification, (**d**) scarring tissue containing asymptomatic macrocalcifications formed in fat necrosis foci, and (**e**) a single example of clearly palpable and symptomatic massively calcified fat necrosis.

All patients were investigated to have tumor bed hardening reported in 19/57 (33.3%), with 9 in group A and 10 in group B (Table 3). Of them, 15 patients (79%) had good to excellent cosmesis, the cosmesis of 3 patients was satisfactory, and 1 patient faced poor cosmetic outcome along with palpable and painful tumor bed (see above in Section 3.2, and late findings on mammography in Figure 4). 

Foci of fat necrosis were identified on mammograms of six patients and magnetic resonance of one (4 in group A, 3 in B). Example mammograms of our patients with no radiological treatment sequelae (a), scarring changes (b), scarring changes with tiny benign calcifications (c), scarring changes with asymptomatic fat necrosis-related macrocalcifications (d), and a case of symptomatic massive fat necrosis (e) are listed in Table 3 and presented in Figure 4.

As mentioned, patients were preselected for HT treatment regarding breast appearance (size and volume). The 3D-treatment plans dose-volume parameters analysis revealed a statistically significant difference between irradiated volumes in group A (29.9 ccs) and B (52.8 ccs), *p* = 0.02. Even though the thermally boosted cohort had larger volumes heated and irradiated, we did not find a difference in late sequelae incidence.

Of note, in a single case, grade 2 telangiectasia occurred (group A). 

Overall, the above-listed differences between both groups appeared to be not statistically significant. 

## 4. Discussion

The study presents long-term oncological results with the incidence of adverse effects in a unique postoperative adjuvant setting. Combined irradiation of surgically conserved breast consisted of EBRT, and the thermally augmented interstitial HDR-BT boost was investigated. From the literature, the combination of RT with HT is firmly justified in scenarios of recurrent, locally advanced, or inoperable BC [15,17,26,27]. Here, the treatment results relate to unusual HT application combined with mandatory postoperative tumor bed irradiation [20,21,22].

Following the concept presented by Dooley et al. [28], who suggested achieving maximum positive margins incidence reduction in early BC patients focally heated before mastectomy, we aimed to thermally enhance HDR-BT boost efficacy in tumor bed potentially invasive cells eradication. Some years earlier, in 1997, Hartmann et al.’s study resulted in about 50% BCT feasibility after preoperative BC (for tumors larger than 3 cm at presentation) treatment combined with chemotherapy, EBRT, and, similarly to ours, preceding 10 Gy interstitial BT boost [29]. However, a relatively short 20-month follow-up makes further comparison difficult. Some more preoperative attempts of thermotherapy for BC were undertaken. They were often based on focused MW external systems and thus are not discussed in detail [30,31,32,33].

A current review published in 2020 by Kok et al. maintains that for breast, head and neck, or prostate malignancies, heating technologies, such as local interstitial MWHT, are still successfully utilized for radiosensitization [34]. As reported by Arunachalam et al., almost simultaneous thermobrachytherapy (HTRT) of BC chest wall recurrences may be characterized by as high as a 2.5 thermal enhancement ratio (TER) [35]. The rationale for that was recently raised by Datta et al. They attempted to calculate the HT-related breast tumor α/β ratio for 259 recurrent BC patients treated in three trials and estimated it between 1.89 and 2.15 [18,27,36]. That means hypofractionated irradiation regimens (e.g., high doses of HDR-BT) are even more justified and have the potential for more effective tumorous cells eradication compared to solitary and standardly fractionated protocols.

Our original personalized offer for BC patients treated conservatively was feasible and well-tolerated. All eligible patients were willing to have the interstitial BT boost enhanced by safely delivering heat to their tumor beds. We found no similar reports on heating potentially cancerous cells-containing breast tissue regarding efficacy and toxicity. Of note, almost three decades ago, Vernon et al. showed a clear LC improvement in BC treatment consisting of RT and HT. The summary of five randomized trials failed to prove a benefit in overall survival. Still, it delivered some data on the safety of local HT in terms of early and late toxicity [27]. However, it must be remembered that exceeding temperatures of 44–45.0 °C or higher may affect healthy tissues and result in pain, superficial burns, sores, or soft tissue necrosis [14,28,37,38,39]. After Datta et al., about recurrent BC HTRT, combined treatment increases locoregional control probability by 22% up to two-thirds and causes minimal acute and late toxicities [40].

It was mentioned earlier that a higher dose administered to the BC tumor bed reduces the 5-year LRR from 7.3–13.3% to 3.6–6.3% [6,7,8,10]. In the study, 5-year and 10-year LC reached 100% and 96%, respectively, irrespective of the group. Initially, it was expected to report a higher incidence of local recurrences. In the treated population, both EBRT plus HDR-BT boost combinations with or without HT appeared to be highly effective; thus, the advantage of adding HT could not be identified. This phenomenon was noticed quite quickly during the data collection. Subsequently, MWHT was offered only to patients with high-risk features: young patients’ age (<50 years), high-grade invasive tumors (G3), adjacent to invasive ductal carcinoma in situ (DCIS), positive nodal status, close or positive surgical margins, indications for chemotherapy, perineural invasion (PNI), lymphovascular invasion (LVI), triple-negative BC (TNBC), and HER2-positive BC. This decision is in concordance with the EORTC Boost vs. No Boost Trial conclusions updated in 2017 by Vrieling et al. They proved that in high-risk young patients with adjacent DCIS 20-year ipsilateral breast tumor recurrence (IBTR) could be decreased by the boost from very high 31% to moderate 15% (*p* < 0.001) [9]. Based on that, we are convinced that it is worth making efforts to further improve treatment efficacy in this selected patient cohort [41,42,43,44,45,46]. Our significant group of subsequent high-risk patients is now being evaluated, and the treatment efficacy and late toxicity results are to be presented soon.

The achieved cosmetic outcome is comparable to other studies implementing interstitial BT boost. Guinot et al. [45], Serkies et al. [47], Dolezel et al. [48], or Quéro et al. [44] reported on good to excellent CO in 97%, 91%, 82.6%, and 80%, respectively. Here we can provide data on good to excellent CO in 96% (group A) and 87.5% (group B), *p* = 0.68. 

Clinically, in one-third of patients, some persisting and palpable tumor bed hardening was noted. It also has to be mentioned that a great proportion of these findings were already present before BT as a result of surgery. Understandably, further irradiation adds to the risk of fibrosis in treated volume. However, one-fifth of our patients had no changes in control mammograms, 40.4% had slight scarring (increased density), 21% scarring with benign macrocalcifications, and in seven patients (12.3%) fat necrosis developed, including one symptomatic case. Fat necrosis (FN) is a form of mild injury to the tissue manifesting in radiolucent areas. In time, they might become rimmed with calcifications. FN incidence differs depending on the available scarce reports that cannot be directly compared to our results because of different techniques, primarily balloon-based BT [49,50,51,52].

We identify some weaknesses of the study. The patients’ cohort is a relatively limited group and analyzed retrospectively. All patients were subjectively selected for additional HT treatment. Nevertheless, the only personalized approach based on meticulous individuals’ presentations could be used to provide safe and good-quality TB sessions.

## 5. Conclusions

HDR-BT boost with or without the TB for selected early-stage BC patients treated with BCT was feasible and well-tolerated. The groups distributed all late sequelae, LC, DFS, OS, and CO equally. The TB did not add to late toxicity. The TB advantage could not be identified due to both groups’ high 5- and 10-year LC. The results suggest a study continuation only in high-risk BC patients burdened with a substantially increased risk of local recurrence. The risk and late toxicity profile of adjuvant HT is not an issue.

## Figures and Tables

**Table 1:** Patient and tumor clinical characteristics.

Feature	HDR-BT Alone Group A	HDR-BT + Thermal Boost Group B
**Age, median (range) years**	53 (32–71)	*p* = 0.47 *
≤40	4 (16.0%)	1 (3.1%)
41–50	5 (20.0%)	8 (25.0%)
51–60	9 (36.0%)	13 (40.6%)
≥61	7 (28.0%)	10 (31.3%)
**T stage**		*p* = 0.53 *
T1a	0 (0.0%)	2 (6.3%)
T1b	6 (24.0%)	5 (15.6%)
T1c	16 (64.0%)	17 (53.1%)
T2	2 (8.0%)	6 (18.8%)
Tx	1 (4.0%)	2 (6.3%)
**N stage**		*p* = 0.95 *
N0	17 (68.0%)	20 (62.5%)
N1	5 (20.0%)	12 (37.5%)
N2a	3 (12.0%)	0 (0.0%)
**Clinical stage**		*p* = 0.52 *
I	14 (56.0%)	14 (43.8%)
IIA	7 (28.0%)	14 (43.8%)
IIB	0 (0.0%)	2 (6.3%)
IIIA	3 (12.0%)	0 (0.0%)
n.d.	1 (4.0%)	2 (6.3%)
**Histology**		^ *p* = 0.15
Ductal invasive carcinoma	21 (84.0%)	26 (81.3%)
Lobular invasive carcinoma	2 (8.0%)	0 (0.0%)
Tubular carcinoma	1 (4.0%)	3 (9.4%)
Not specified (post-chemo)	1 (4.0%)	3 (9.4%)
**Grade**		*p* = 0.34 *
G1	9 (36.0%)	11 (34.4%)
G2	13 (52%)	11 (34.4%)
G3	3 (12.0%)	7 (21.9%)
Not specified (post-chemo)	0 (0.0%)	3 (9.4%)
**Estrogen receptor status**		^ *p* = 0.29
ER (+)	18 (72.0%)	21 (65.6%)
ER (−)	7 (28.0%)	8 (25.0%)
n.d.	0 (0.0%)	3 (9.4%)
**Progesteron receptor status**		^ *p* = 0.27
PgR (+)	16 (64.0%)	20 (62.5%)
PgR (−)	9 (36.0%)	9 (28.1%)
n.d.	0 (0.0%)	3 (9.4%)
**HER2 status ^†^**		^ *p* = 0.13
Positive (+)	0 (0.0%)	1 (3.1%)
Negative (−)	22 (88.0%)	21 (65.6%)
n.d.	3 (12.0%) ^†^	10 (31.3%) ^†^
**Lymph node treatment**		*^ p = 0.85*
ALND	23 (92.0%)	29 (90.6%)
SNB ^‡^	2 (8.0%)	3 (9.4%)
**Chemotherapy treatment**		^ *p* = 0.30
Yes	13 (52.0%)	21 (65.6%)
No	12 (48.0%)	11 (34.4%)
**External Beam RT regimen**		*p* = 0.66 *
42.5 Gy/2.5 Gy/17 fx	15 (60.0%)	20 (62.5%)
45.0 Gy/2.25 Gy/20 fx	7 (28.0%)	12 (37.5%)
50.0 Gy/2.0 Gy/25 fx	3 (12.0%)	0 (0.0%)

^ test Chi^2^; * test M-U; ^†^ at the time of the study, HER2 status was not standardly assessed for all patients, and Ki67 status as well; ^‡^ at the time of the study, SNB was not a standard yet. Abbreviations: n.d.—no data; ALND—axillary lymph node dissection; SNB—sentinel node biopsy; RT—radiotherapy; fx—fractions.

**Table 2:** Long-term clinical result.

Feature	HDR-BT Alone Group A	HDR-BT + Thermal Boost Group B
**Overall survival**		*Test log rank*
Alive 46 (80.7%)	19 (76.0%)	27 (84.4%)
Dead 11 (19.3%)	6 (24.0%)	5 (15.6%)
**Local control**		*Test log rank*
Yes 55 (96.5%)	24 (96.0%)	31 (96.9%)
No 2 (3.5%)	1 (4.0%)	1 (3.1%)
**Distant metastases**		*Test log rank*
No 50 (87.7%)	22 (88.0%)	28 (87.5%)
Yes 7 (12.3%)	3 (12.0%)	4 (12.5%)

**Table 3:** Long-term cosmetic outcome, late toxicity, and control mammography findings.

Feature	HDR-BT Alone Group A	HDR-BT + Thermal Boost Group B
**Cosmetic effect**		^ *p* = 0.68
Excellent 32 (56.1%)	15 (60.0%)	17 (53.1%)
Good 20 (35.1%)	9 (36.0%)	11 (34.4%)
Satisfactory 4 (7.0%)	1 (4.0%)	3 (9.4%)
Poor 1 (1.8%)	0 (0.0%)	1 (3.1%)
**Tumor bed hardening**		*^ p* = 0.70
No 38 (66.7%)	16 (64.0%)	22 (68.7%)
Yes 19 (33.3%)	9 (36.0%)	10 (31.3%)
**Teleangiectases**		
No 56 (98.2%)	24 (96.0%)	23 (100.0%)
Yes 1 (1.8%)	1 (4.0%)	0 (0.0%)
**Mammography findings**		^ *p* = 0.34
No changes	7 (28.0%)	5 (15.6%)
In breast scarring	10 (40.0%)	13 (40.7%)
Scarring with calcifications	3 (12.0%)	9 (28.2%)
Asymptomatic fat necrosis	4 (16.0%)	2 (6.2%)
Symptomatic fat necrosis	0 (0.0%)	1 (3.1%)
n.d.	1 (4.0%)	2 (6.2%)

^ test Chi^2^; n.d.—no data.

## Data Availability

Not applicable.
